# Chiral Mesoporous Silica Materials: A Review on Synthetic Strategies and Applications

**DOI:** 10.3390/molecules25173899

**Published:** 2020-08-27

**Authors:** Mingshu Cui, Wei Zhang, Luyao Xie, Lu Chen, Lu Xu

**Affiliations:** School of Pharmacy, Shenyang Pharmaceutical University, Shenyang 110016, China; cui_mingshu@126.com (M.C.); zhangwei9501@126.com (W.Z.); xieluyao0221@163.com (L.X.); chenlu182125@163.com (L.C.)

**Keywords:** chirality, mesoporous silica, synthesis, application

## Abstract

Because of its tunable textural properties and chirality feature, chiral mesoporous silica (CMS) gained significant consideration in many fields and has been developed rapidly in recent years. In this review, we provide an overview of synthesis strategies for fabricating CMS together with its main applications. The properties of CMS, including morphology and mesostructures and enantiomer excess (ee), can be altered according to the synthetic conditions during the synthesis process. Despite its primary stage, CMS has attracted extensive attention in many fields. In particular, CMS nanoparticles are widely used for enantioselective resolution and adsorption of chiral compounds with desirable separation capability. Also, CMS acts as a promising candidate for the effective delivery of chiral or achiral drugs to produce a chiral-responsive manner. Moreover, CMS also plays an important role in chromatographic separations and asymmetric catalysis. There has been an in-depth review of the synthetic methods and mechanisms of CMS. And this review aims to give a deep insight into the synthesis and application of CMS, especially in recent years, and highlights the significance that it may have in the future.

## 1. Introduction

Chirality is a universal feature of the universe, embodied in the generation and evolution of life. In 1848, chemists first came up with the definition of isomers in the study of tartaric acid, which led to the concept of chirality. The so-called chirality refers to the feature that the object and its mirror image cannot overlap exactly, which can also be explained as the lack of S_n_ symmetry element. Enantiomers almost share the same physical properties (melting point, boiling point, etc.), thermodynamic properties (free energy, enthalpy, entropy, etc.), and chemical properties. While in a chiral environment (e.g., a chiral reagent, a chiral solvent), a difference in physic-chemical properties appears.

In biology, chirality can be found in a variety of biomolecules, including amino acids, sugars, proteins, and DNA. It is widely acknowledged that many functions of living organisms are performed by proteins, which are made up of amino acids. Of the twenty amino acids that make up life, except for glycine, which is not chiral, the other nineteen are almost always left-handed. The sugars in living organisms, however, are all right-handed, including deoxyribose. Thus, naturally produced DNA with transcriptional activity is mainly dextrorotatory. Vines of plants, organisms such as conches, and even microorganisms themselves also possess chiral structures. Nevertheless, if the compounds with stereoisomerism happen to be drugs then it may result in significant differences in pharmacology, metabolic process, toxicity, and efficacy of different enantiomers in the human body. Other fields such as mechanical engineering also face the mystery of chirality. For example, helical springs reversibly store energy by elastic deformation of wires to distribute the induced strain evenly. Now chirality describes not only the symmetry of objects and molecules but also inorganic nanostructures. Chiral nanostructures have attracted much attention due to their favorable interactions with chiral molecules, and many researchers have been devoted to the synthesis and formation mechanism research of chiral nanostructures.

Chiral assemblies can be classified by various methods, and a typical classification is based on the inorganic raw materials for synthesis, including chiral silica nanostructures [1,2,3,4,5,6], chiral metal nanostructures [7,8], chiral carbon nanomaterials [9,10,11], chiral semiconductor nanoparticles [12,13,14], and other nanomaterials [15,16]. One of the general approaches for the preparation of chiral nanostructures is through the assembly of chiral templates or chiral building blocks and organic molecules such as amino acids and sugars, some of which are called biomimetic synthesis due to the similarities between these initial units and the biomolecules [17]. Besides, the assembly of achiral basic units into well-organized nanostructures in specific handedness is another important design. When it comes to the synthesis of chiral nanostructures, CMS probably is one of the most common examples. Generally, typical mesoporous silica can be obtained via the self-assembly of amphiphiles and inorganic silica sources since its first discovery in the 1990s by the Mobile Oil Corporation. Since then, mesoporous silica has come into focus due to its superior properties such as tunable textural properties [18,19,20], biocompatibility [21,22], biodegradability [23,24,25], etc. There have been considerable reports available for the synthesis, functionalization, and application of mesoporous silica [26,27,28,29], and numerous researches are still underway. Based on mesoporous silica, chiral mesoporous silica is endowed with chirality due to the chiral modification or the existence of helical channels, which broadens its applications in areas such as drug delivery, chiral separation of biomolecules, and asymmetric catalysis, although some of these are still in initial stages.

## 2. Synthesis of Chiral Mesoporous Silica (CMS) Nanoparticles

**Figure 1:**
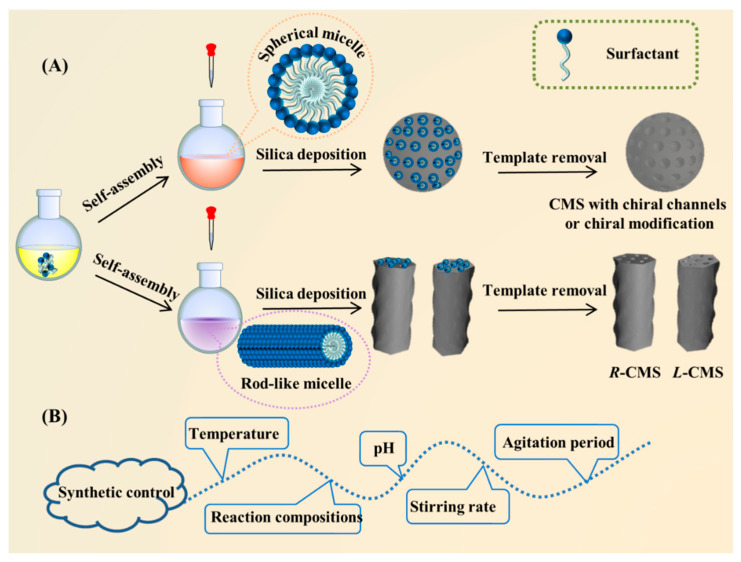
(**A**) Schematic diagram of CMS synthesis by surfactants; (**B**) Factors contributing to the synthetic control of CMS. Credit: original figure.

CMS nanoparticles are mainly prepared via cooperative self-assembly of chiral or achiral amphiphiles and silica precursors, as well as other methods (Table 1). The typical synthetic pathways of CMS in the presence of surfactants are illustrated in Figure 1, and the specific strategies are discussed in the following sections.

### 2.1. CMS Templated by Chiral Anionic Surfactants

CMS material was firstly discovered by using amino acid-derived chiral anionic template *N*-myristoyl-l-alanine and *N*-trimethoxysilylpropyl-*N*,*N*,*N*-trimethylammonium chloride (TMAPS) or 3-aminopropyltrimethoxysilane (APTES) as a co-structure-directing agent (CSDA) [30]. The assembly of anionic surfactant and silicate is assisted by CSDA via electrostatic interactions. The positively charged ammonium ion and the alkoxysilane sites of APS or TMAPS interact with the negatively charged head group of anionic amphiphile and tetraethoxysilane (TEOS), respectively [2]. Helical nanotubes [54] and twisted mesoporous silica nanoribbons [55] derived from other amino acid derivatives and silicates via sol-gel transcription process can also be obtained.

**Figure 2:**
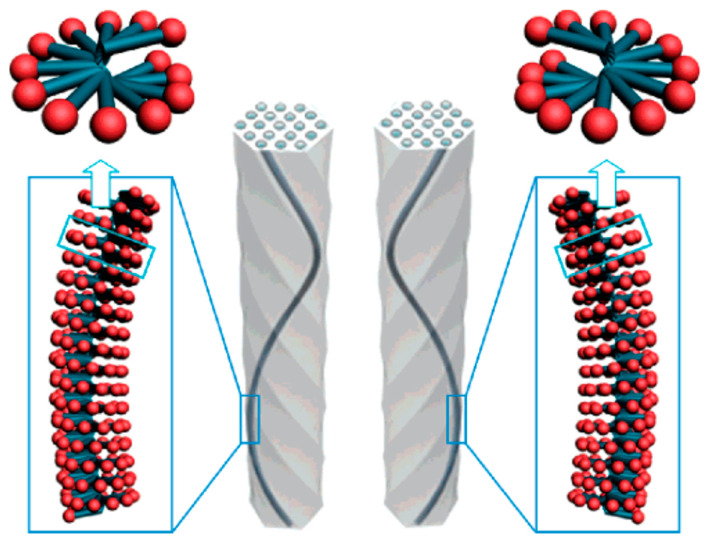
Molecular origin of the left-handed (**left**) and right-handed (**right**) helical structure of the CMS derived from the helical propellerlike packing of the chiral amphiphilic molecules. Credit: reprinted with permission from Reference [37]. Copyright 2008 American Chemical Society.

Structural control has always been a long-term research topic in the field of material science due to its characteristics and diverse application requirements. Similar to traditional mesoporous silica, the control of CMS involves morphology and mesostructures, but the ratio of left-/right-handedness (enantiomer excess, abbreviated as ee) is also a core point of research. Jin et al. synthesized helical CMS via the self-assembling of chiral anionic amphiphiles *N*-acyl-*l*-alanine by CSDA method and further systematically discussed how different anionic ions influenced the morphology, mesostructured, and pore size of the CMS, including Cl^−^, Br^−^, NO_3_^−^, and SO_4_^2−^ [31]. Che et al. demonstrated that the stirring rate can alter the morphology and helicity of the CMS during the self-assembly of chiral surfactant [32]. Stirring rates higher than 400 rpm are conductive to improve the uniformity of mesoporous silica nanorods with different diameters and helical pitches. Unlike the CMS which owes its chirality to helical channels and twisted morphology and helical nanochannel, the chirality of mesoporous silica can also be derived from the silicate building blocks, where achiral templates and a chiral cobalt complex act as co-templates via electrostatic interaction [33]. Yokoi et al. reported predominantly left-handed or right-handed CMS by using C_14_-l-AlaA and C_14_-d-AlaA respectively or in combination. They also confirmed how synthetic conditions including gel compositions, stirring, and agitation periods affected the CMS synthesis greatly [34]. Later work confirmed the important role of chiral dopant and 1,2-bis(triethoxysilyl)ethylene (BTEE) in the controlled synthesis of enantiomerically pure CMS [35]. This research was performed by using a chiral anionic surfactant, C_14_-l-AlaA, as a template together with l-arginine or d-arginine as a chiral dopant. Both right-handed and left-handed CMS (*r*- and *l*-CMS) were produced when only l-enantiomer was employed as the chiral surfactant, proving that the ratio of the *r*- and *l*-CMS was not only governed by the intrinsic chirality of the surfactant template but other factors [30]. In another work, they claimed that ee of the CMSs formed with C_16_-l-Phe was inversely associated with the basicity of the reaction solution [36]. Although the specific mechanism has not been completely disclosed, there is no doubt that the ratio of *r*- and *l*-CMS is controlled either by kinetics or by thermodynamics throughout the synthesis process. By further effort, it was found that both the substituent’s steric bulk and the temperature were key factors influencing the ee of the CMS [37]. The amphiphilic molecules may present helical packing, the handedness of which is dependent on the chirality of the amphiphilic molecule (Figure 2). Based on thermodynamics principle, the ee of the CMS is determined by the relative rate constant for *l*- and *r*-CMS formation or by the relative stability of *l*- and *r*-CMS, and the generation of the diastereomeric conformer can be explained as a result of the rotation of the C_α_-N single bond of the amphiphilic molecule.

Generally, the two rotational isomers reach the equilibration quickly at ambient temperature. However, the equilibration is significantly slowed down due to the stacked micellar structure. Consequently, for the l-form *N*-acylamino acids, the conformer of lower energy led to *l*-CMS when stacking, while the less unstable conformer produced *r*-CMS. As mentioned above, the emergence of a chiral or helical structure in CMS is a confluence of factors that come together to cause this, including the inherent chirality of molecules together with thermodynamic factors such as temperature, stirring rate, and pH (Figure 1B).

### 2.2. CMS Templated by Chiral Cationic Surfactants and Gelators

**Figure 3:**
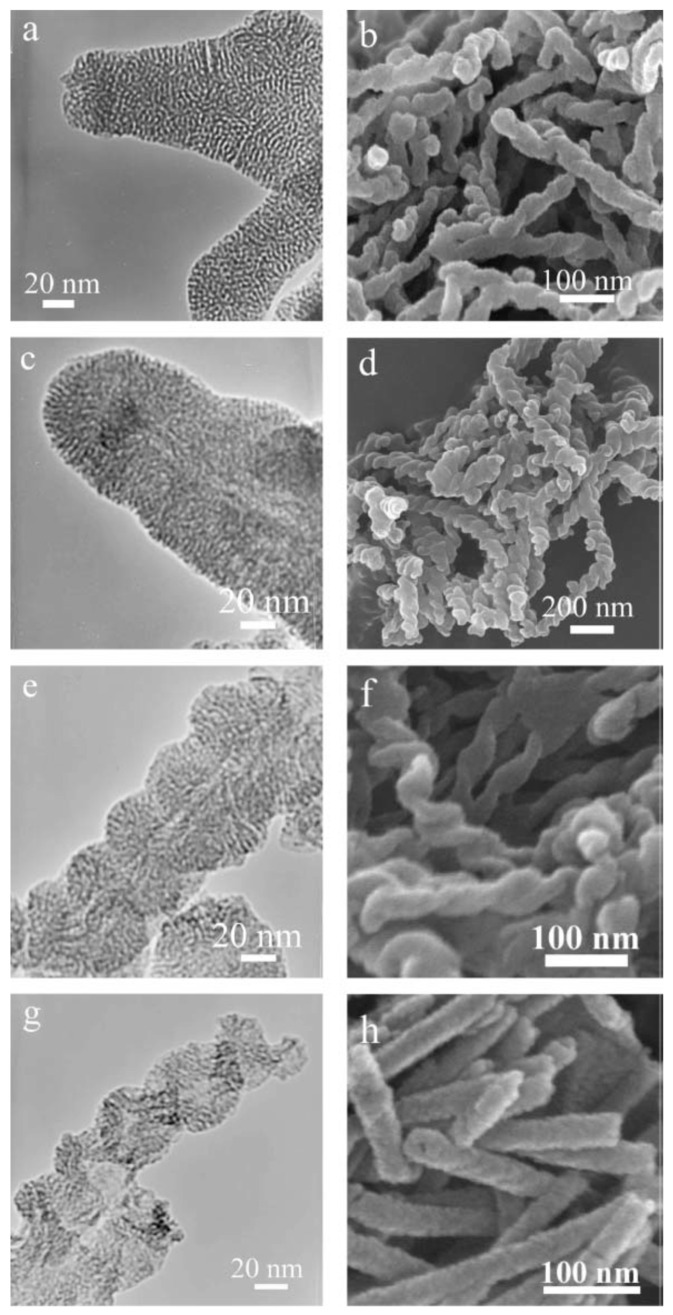
(**a**) TEM image of calcined mesoporous silica nanofibers (preparation condition: (10 mg l-4PyCl: 0.9 mL 1.0 wt% NH_3_ aq.: 0.1 mL ethanol: 20 mg TEOS)); (**b**) SEM and (**c**) TEM images of calcined mesoporous silica nanofibers (Preparation condition: (10 mg l-4PyCl: 0.6 mL 1.0 wt% NH_3_ aq.: 0.4 mL ethanol: 20 mg TEOS)); (**d**,**f**) SEM and (**e**,**g**) TEM images of calcined mesoporous silica nanofibers (preparation condition: (10 mg l-4PyCl: 0.7 mL 11.3 wt%NH_3_ aq.: 0.3 mL ethanol: 20 mg TEOS)). (**h**) SEM image of calcined mesoporous silica nanofibers (Preparation condition: (10 mg l-4PyCl: 0.5 mL 1.0 wt% NH_3_ aq.: 0.5 mL ethanol: 20 mg TEOS)). Credit: republished with permission of Royal Society of Chemistry, from [38], Copyright (2005); permission conveyed through Copyright Clearance Center, Inc.

Aside from the chiral anionic surfactants, chiral cationic surfactants have also been applied to synthesize CMS. Yang et al. successfully prepared helical CMS via sol-gel transcription by using gelators-like cationic surfactants l-4PyCl as a template, which was derived from l-isoleucine [38]. As depicted in Figure 3, the helical structure varied with the concentration of NH_3_, and the helical pitch was correlated reciprocally with the concentration of NH_3_. The structure can be controlled by the volume ratio of water to alcohol. In later work, they obtained helical or twisted mesoporous silica of different pitches by varying the concentration of NH_3_, and the volume ratio of water to alcohol or other solvents [39]. It can be explained that the controllable formation depends on the speed of the silica oligomer’s condensation and the l-4PyCl self-organization. A lower volume ratio of alcohol to water prompted the synthesis of mesoporous silicas because of the resulted faster condensation speed of silica oligomers and slower self-organization speed of l-4PyCl. Otherwise, silica nanotubes or nanoribbons were obtained. Then, Yang’s group prepared helical mesoporous silica nanotubes composed of two left-handed twisted nanoribbons templated by chiral cationic gelators which were derived from l-phenylalanine, l-valine, and l-isoleucine, respectively [40,41]. Take l-PhePyBr (prepared in the presence of l-phenylalanine) as a typical example: the reorganization of l-PhePyBr self-assemblies was closely related to the ethanol hydrolyzed from TEOS and orthosilicate anionic ions. Silica oligomers penetrated the voids between the l-PhePyBr self-assemblies and then polycondensed on the surface after the cationic–anionic ion interactions based on orthosilicate anionic ions and the single organic self-assemblies.

### 2.3. CMS Templated by Achiral Templates

**Figure 4:**
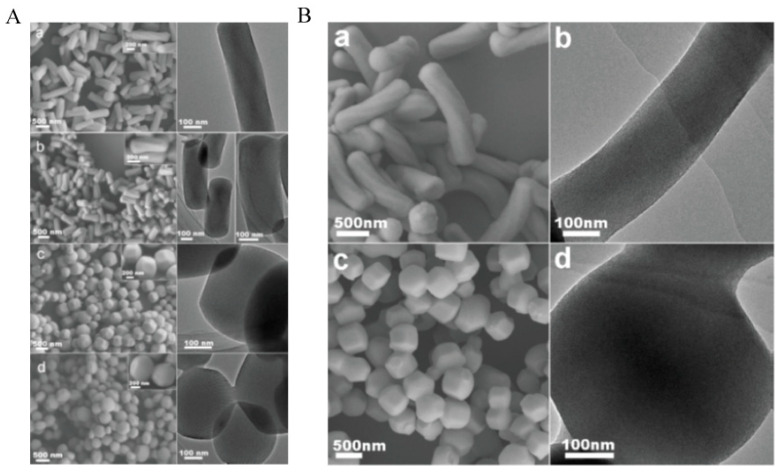
(**A**) SEM images (left), high-magnification SEM images (inset), and TEM images (right) for (**a**) CMS-20, (**b**) CMS-15, (**c**) CMS-10, and (**d**) CMS-5; (**B**): (**a**,**c**) SEM and (**b**,**d**) TEM images of mesoporous silica synthesized with C14TMAB in (**a**,**b**) 25 wt% and (**c**,**d**) 20 wt% ammonia solution. Credit: republished with permission of John Wiley and Sons, from [43], Copyright (2007), permission conveyed through Copyright Clearance Center, Inc.

In contrast to CMS synthesized based on chiral templates, CMS materials can also be prepared by using achiral templates. Lin et al. proposed that the tight intermolecular packing based on long alkyl chains of achiral C_18_MIMBr molecules induced the formation of helical micelles [42]. Besides, CMS was fabricated by a simple system composed of achiral cationic surfactant, and meanwhile, the entropically driven model was introduced to explain the formation mechanism, which was described by the Equation (1):

∆F ∝ 1/2 *Ll_p_k*^2^ − *n*V_overlap_ (1)

where *l_p_* is the length scale over which a rod can bend, *n* is the sphere concentration, ∆F is the bending stiffness, and *r* is the radius of the sphere [43,56]. As shown in Figure 4A, shorter particles (*L*) with less curved channels (*k*) will be formed with decreasing ammonia concentration (*n*), to keep ∆F < 0. Also, rod-like micelles formed with shorter hydrocarbon chains are more rigid and thus difficult to bend into the helix (Figure 4B). Therefore, the ammonia concentration required in the C_14_TMAB system is higher than that of the C_16_TMAB system in order to achieve the same helicity.

**Figure 5:**
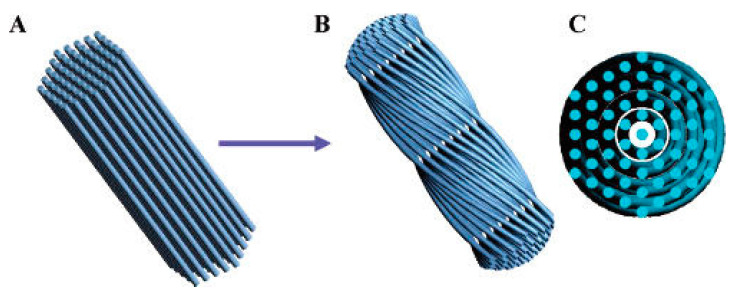
Schematic drawing illustrating the formation of helical mesostructured rods from hexagonally arrayed straight rod-like micelles with equal length (**A**) to a helical rod with two rounded ends viewed perpendicular to the length direction (**B**) and the hexagonal cross-section viewed parallel to the length direction (**C**). Credit: reprinted with permission from [44]. Copyright 2006 American Chemical Society.

Differently, Yang et al. attributed the origin of the helical mesostructured materials as a result of morphological transformation along with the reduction in the surface free energy [44]. They obtained helical CMS in the presence of cetyltrimethylammonium bromide (CTAB) and perfluorooctanoic acid, proposing that the spiral morphology is derived from bundles of straight rod-like composite micelles with equal length rather than a single rod-like micelle (Figure 5).

Helical mesoporous silica nanorods can also be successfully synthesized using CTAB as a template and achiral alcohols as CSDA, where the formation of the helix was driven by the reduction in surface free energy [45]. This is based on the fact that higher molarity of alcohol contributes to the transformation of spherical micelles to rod-like and worm-like micelles. Long alkyl chains of alkyl alcohols decrease the critical micelle concentrations of CTAB in aqueous solution. Likewise, twisted rod-like CMS prepared with CTAB and *n*-heptanol or *n*-nonanol also demonstrated the significance of the molar ratio of alcohol/CTAB and chain length of alcohols for the generation of chirality [46]. In another work, Gao et al. reported a facile method to prepare CMS by using CTAB as a template without any other additives [47]. They suggested that the morphology can be controlled by varying the concentration of the ammonia solution.

Although there is no certain mechanism for the synthesis of CMS via achiral templating process, what we can confirm from the above results is that molecular chirality is not the only determinant; that is, there are other driving forces. Moreover, this also puts forward the new expectations for the follow-up research of CMS, which is to elaborate on the concrete mechanism of its synthesis.

### 2.4. CMS with Molecular Chirality

**Figure 6:**
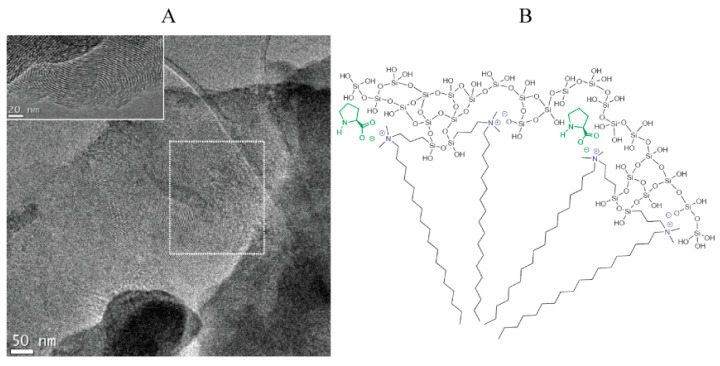
(**A**) TEM micrograph of the calcined material showing the different orientation of the mesopores, which may induce the chirality. The “eight-like” domain is marked by the dashed square, while the inset corresponds to a different particle. (**B**) The sector of the suggested micelle constituted by alternating C_18_-TMS dimers and amino acid (proline) molecules or negatively charged silica species. The organization of the aminosilane dimers into micelles serves as a template for the formation of the ordered mesoporous silica, while the amino acid transfers its chirality to the micelle and from there to the silica. Credit: reprinted with permission from [48]. Copyright 2011 American Chemical Society.

The molecular imprinting technique acts as a methodology for the formation of specific cavities to selectively bind ‘guest’ molecules in the presence of templates [41]. These resulting molecularly imprinted materials are characterized by a high degree of stereo- and regiospecific selectivity, offering enormous potential for chiral separation [57,58,59,60,61] and antibody mimics [62,63,64,65]. It has been logical, therefore, to apply chiral imprinting in the design of CMS with amino acids [48]. Figure 6A confirmed the efficient transference of chirality from amino acid to the ordered mesoporous silica. The formation was hypothesized to be driven by the electrostatic interaction between amino acid molecules and positively charged *N*-3[3-(trimethoxysilyl)propyl]-*N*-octadecyl-*N*,*N*-dimethylammonium chloride (C_18_-TMS) dimers together with the covalent bonds between hydrolyzed C_18_-TMS surfactant molecules and silanol groups of silicate species (Figure 6B). Su et al. obtained l-phenylalanine imprinted CMS in the presence of phenylalanine and speculated that electrostatic interaction existed between the negatively charged amino acid molecules and the positively charged C_18_-TMS that formed the micelles. Also, the silica skeleton was formed by covalent bonds between C_18_-TMS micelles and silica species. Afterward, the chirality was effectively transferred from the amino acid to the silica [49].

CMS with molecular chirality can also be fabricated by grafting molecular functional groups. Li et al. successfully synthesized spherical CMS nanoparticles with concealed pore channels by grafting l-tartaric acid or d-tartaric acid, respectively [50,51]. Considering the defect of reducing pore space due to the grafting group, Guo et al. designed enlarged pore CMS nanoparticles via the introduction of pore-enlarging agent 1,3,5-trimethylbenzene (TMB) while functional groups were grafted [52,53]. This provides more possibilities for further modification of CMS via grafting groups.

## 3. Applications of Chiral Mesoporous Silica

**Figure 7:**
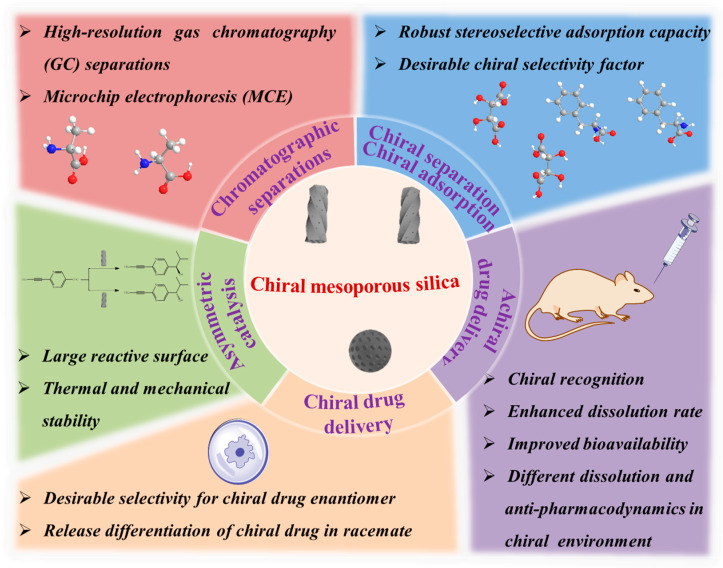
Various applications of CMS. Credit: original figure.

The excellent properties of chiral nanomaterials have triggered their multi-field applications and evaluation [66,67,68,69]. Unique structural features make CMS attractive for various purposes (Figure 7 and Table 2), and the following sections discuss their applications in these specific fields.

### 3.1. Enantioselective Resolution and Adsorption

As the demand for pure enantiomers increases significantly, it is urgent to provide efficient strategies for analytical and preparative separations of enantiomers. To date, a series of effective techniques for the resolution of enantiomers have been developed [70,71,72,73]. CMS material presents unique chiral-structural features and desirable stereoselective adsorption capacity, making it an emerging candidate for chiral adsorption and enantiomeric separation. As proved by circular dichroism (CD) spectroscopy, monodispersed CMS spheres with chiral pores showed the asymmetric preferential adsorption of alanine with a chiral selectivity factor of 3.15 [74]. Interestingly, it was found that the adsorption of d-proline by CMS synthesized with d-proline was higher than that of l-proline, while the adsorption of l-proline by CMS synthesized with l-proline was significantly higher than that of d-proline [75]. This adsorption difference could be ascribed to the imprinting left by the amino acid during the preparation process. CMS templated by chiral block copolymers was demonstrated to be effective in the preferable adsorption of a specific enantiomer of valine with a desirable chiral selectivity factor [76,77]. Previously reported results proved that some chiral sol-gel silica materials with various chiral entities lost the enantioselective preferences after surfactant removal [78,79]. However, the calcined CMS prepared with l-proline still exhibited favorable potential for enantiomeric separation in the resolution of racemic mixtures [48]. This could be explained by the chiral and quite rigid pyrrolidine ring of proline. The imprinting chirality of CMS can also make it work in stereoselective adsorption of l- and d-phenylalanine with a chiral selective factor of 3.24 [49]. The chiral transcription of guanosine monophosphate and folic acid onto mesoporous silica is disclosed via the kinetic adsorption of enantiomeric pairs including *d*-(−)-Tartaric acid and *l*-(+)-Tartaric acid, *l*-Valine, and *d*-Valine, as well as (+)-a-Pinene and (−)-a-Pinene [80]. The two samples exhibited opposite enantiomeric selectivity for enantiomeric pairs due to the chiral transcription process. Therefore, CMS shows a promising prospect in the field of chiral adsorption and separation.

### 3.2. Chromatographic Separations

As effective instrumental techniques for the separation of chiral enantiomers, high-performance liquid chromatography (HPLC) [81], gas chromatography (GC), thin-layer chromatography (TLC) [82] and capillary electrophoresis (CE) make great contributions and account for a great proportion [83,84,85]. Owing to the nematic structure, chirality, and large pore size of chiral nematic mesoporous silica (CNMS), it attracted considerable attention as a chiral stationary phase. An example is the enantioseparation of chiral amino acids using microchip electrophoresis (MCE), where CNMS acted as the chiral stationary phase and hydroxypropyl-β-cyclodextrin was selected as the chiral selector [86]. Besides, CNMS derived from nanocrystalline cellulose shows desirable separation performance in GC or HPLC. As a stationary phase for high-resolution GC, it can give good selectivity for the separation of many achiral compounds and enantiomeric pairs [87]. It also worked as a stationary phase for HPLC, which showed better selectivity for separation of positional isomers when compared with commercial β-cyclodextrin HPLC columns [88].

Owing to the thermal and mechanical stability together with the high selectivity of CMS, it may be a favorable strategy for the high-resolution gas chromatographic separations. Yuan group was the pioneer in applying highly ordered CMS as a stationary phase for high-resolution GC separations, which presented excellent selectivity for the separation of many organic compounds [89]. Later, they reported a CMS-coated open tubular column for the separation of eighteen racemates. The CMS was prepared via the self-assembly of sodium dodecyl sulfate (SDS) together with chiral amino alcohols [90,91]. It not only showed excellent selectivity but short analysis times and high-temperature resistance. To sum up, CMS has a broad prospect as a stationary phase in chromatographic analysis, which will be of great help for the resolution of compounds.

### 3.3. Chiral Drug Delivery

**Figure 8:**
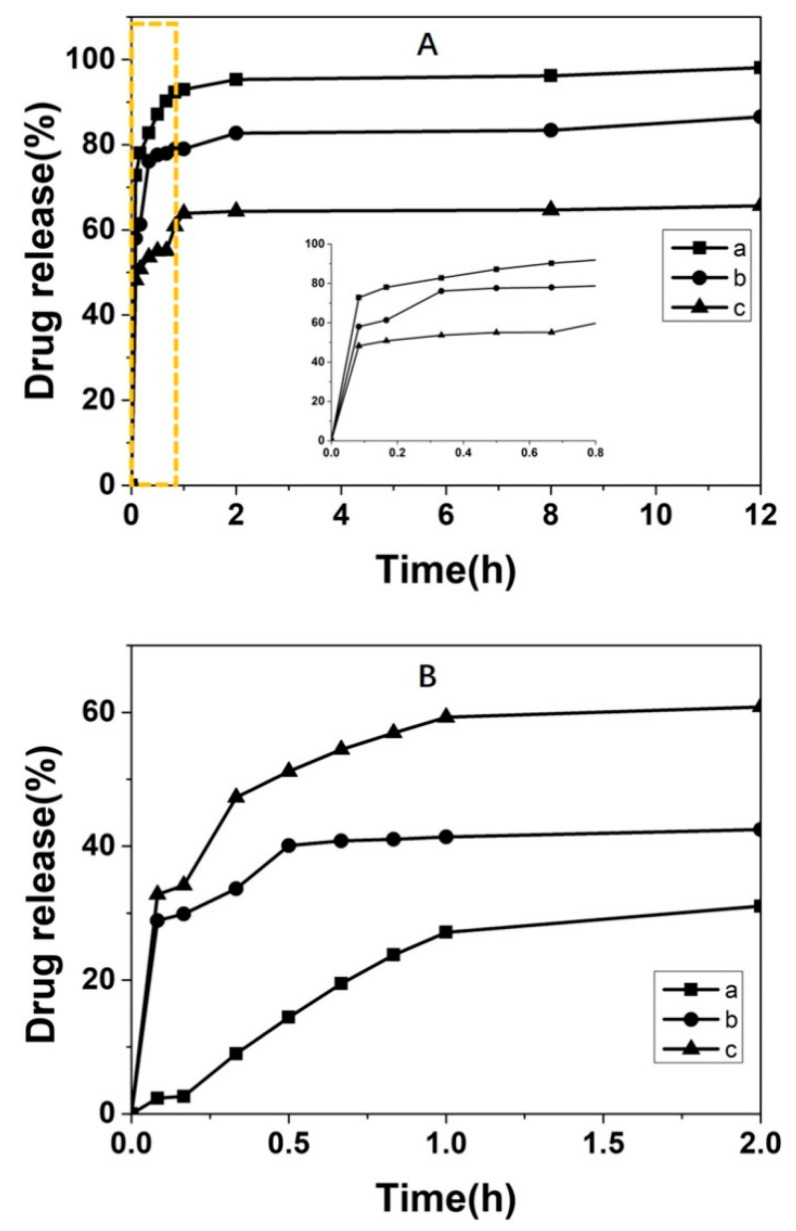
In vitro release profiles of (**A**): (**a**) IBU, (**b**) IBU loaded l-CMS, (**c**) IBU loaded d-CMS in simulated intestinal fluid (SIF) (the inset figure is the enlargement of the rectangle area labeled in Figure (**A**); (**B**): (**a**) IBU, (**b**) IBU loaded l-CMS, (**c**) IBU loaded d-CMS in simulated gastric fluid (SGF). Credit: republished with permission of Elsevier, from Reference [104], Copyright (2019), permission conveyed through Copyright Clearance Center, Inc.

Mesoporous silica has been widely utilized as a drug carrier in drug delivery since its first report in 2001 [92,93,94,95,96] along with many other nanoparticles [97,98,99,100]. Despite the presence of many identical physical and chemical properties in a pair of chiral drugs under achiral conditions, differences will emerge when it comes to the chiral environment of the human body, mainly in the pharmacological activity and toxicological effects. In other words, one isomer may exert the expected active effects, while the other may be inactive or even produce toxic effects. In the pharmaceutical field, chiral drugs account for more than 50% of the total number of drugs according to statistics [101]. Take ofloxacin, for instance; it is a fluoroquinolone antibiotic against both gram-negative and gram-positive bacteria, and levorotatory ofloxacin exerts superior pharmacological activity than the dextral form. Picenadol is an opioid mixed agonist-antagonist for the relief of pain, whose *d*-optical isomer acts as an opioid agonist, while the *l*-isomer is an opioid antagonist [102]. Another example is that thalidomide was a major teratogen that caused birth defects in the 1960s. It was found that the (*R*)-thalidomide inhibited the reaction of pregnancy, while the (*S*)-thalidomide could lead to miscarriage and even teratogenicity of newborns. Therefore, it is of great practical significance to promote the in-depth study of chiral drugs in the pharmaceutical field as well as for in-clinic application. Therefore, the rational use of chiral drugs not only contributes to improving pharmacological activity but reducing side effects. The emergence of CMS paved a new way for the effective delivery of chiral drugs. As demonstrated in experiments, chiral mesoporous silica can exhibit desirable selectivity for chiral drugs (metoprolol) due to the preferential binding of one enantiomer over that of the other to the asymmetric specific binding sites in CMS, without any other chemical modifications [103]. Another such study was reported by Xu’s group, where levofloxacin was loaded into the CMS with opposite chirality, demonstrating the difference in drug release [46]. Wang et al. successfully designed twisted rod-like CMS by using l- and d-alanine derivatives as templates for the release differentiation of ibuprofen in simulated intestinal fluid and simulated gastric fluid (Figure 8) on account of the ionization of ibuprofen and the effect of pore geometry on the drug loading and dissolution process [104,105]. Therefore, CMS is promising to be carriers not only for the preferential delivery of enantiomerically pure drugs but for the enantioselectively controlled release of racemic mixtures by the combination of mesoporous structure and helical channels or chiral modification in one CMS nanoparticle.

### 3.4. Achiral Drug Delivery

CMS has also been employed as a drug carrier to adjust and enhance the release of achiral drugs. When CMS with different morphology and helicity but similar pore sizes were utilized as drug carriers for aspirin and indomethacin (IMC), variable release profiles were observed, where longer and more twisted diffusion channels led to a slower release rate [106,107]. After being loaded into the CMS, the improved drug dissolution and a significant rapid release of curcumin (poorly water-soluble drug) under different dissolution media can be accomplished due to the crystalline-to-amorphous transformation [47].

**Figure 9:**
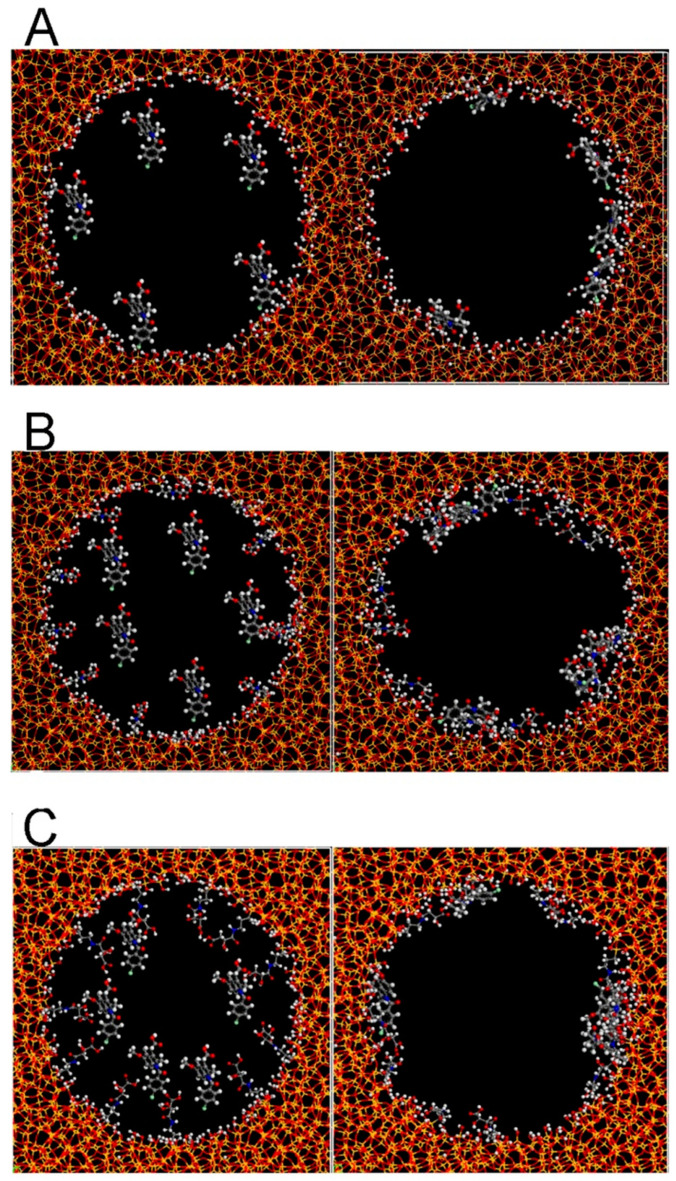
Molecular Simulation results of (**A**), IMC loaded *N*-MSN; (**B**), IMC loaded Cb-d-MSN; (**C**), IMC loaded Cb-l-MSN. Credit: republished with permission of Elsevier, from Reference [50], Copyright (2018), with permission conveyed through Copyright Clearance Center, Inc.

Furthermore, it was disclosed that the chirality-responsive drug delivery systems can be constructed to endow achiral drugs with specific selectivity in the simulated chiral environment to exert functions. When CMS templated by histidine-derivative (C_16_-l-histidine) via biomimetic synthesis was employed as a drug carrier for nimodipine (poorly water-soluble drug), it can significantly improve the dissolution rate and bioavailability as well as the brain distribution in vivo [108]. Li et al. successfully prepared the concealed body mesoporous silica nanoparticles that exerted different chiral recognition functions for the delivery of (IMC) [50]. IMC loaded Cb-d-MSN and IMC loaded Cb-l-MSN showed quite similar dissolution behaviors in enzyme-free simulated intestinal fluid. However, an obvious difference can be seen in plasma drug concentration profiles after oral administration: the C_max_ of Cb-d-MSN was much higher than that of IMC loaded Cb-l-MSN. This could be explained by the molecular simulation results as shown in Figure 9, where the adsorbed IMC was distributed in these two carriers differently as a result of the chiral modification and pore properties. Their later work fabricated On-Off CMS nanoparticles for delivering IMC in the chiral environment and owed the differences in dissolution and anti-pharmacodynamics to on or off chiral recognition functions [51].

**Figure 10:**
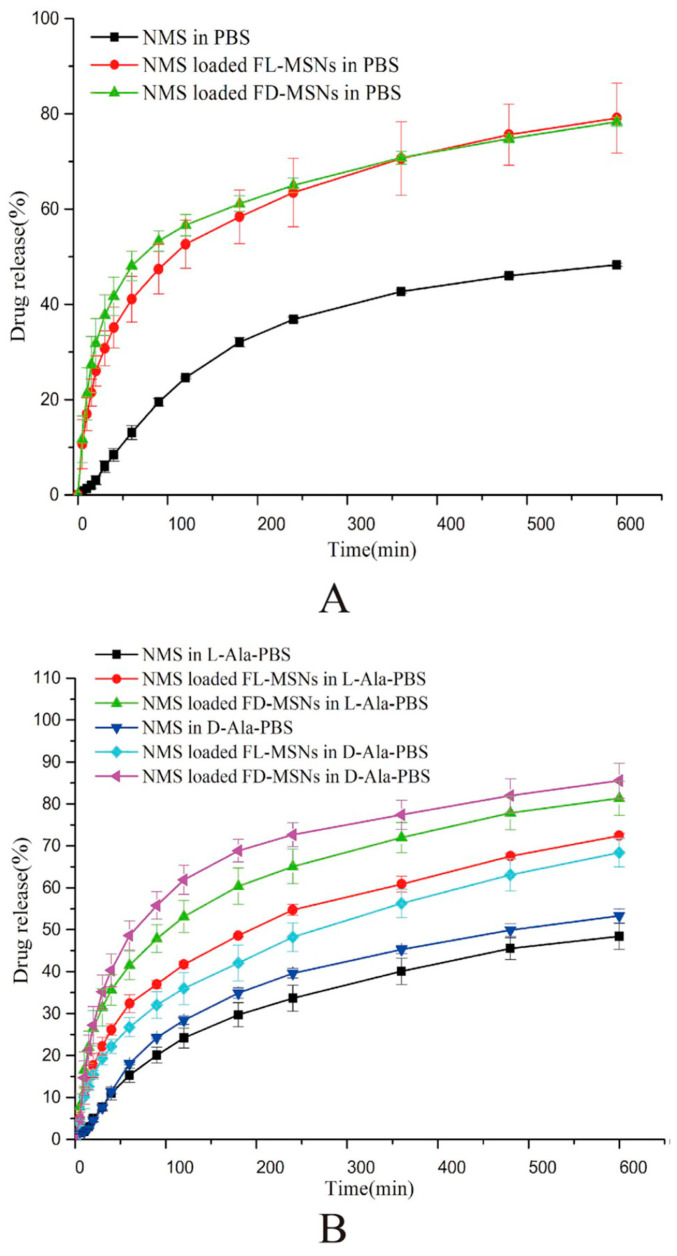
(**A**) In vitro release profiles of NMS, NMS loaded FL-MSNs, and NMS loaded FD-MSNs in pH 6.8 PBS. (**B**) In vitro release profiles of NMS, NMS loaded FL-MSNs, and NMS loaded FD-MSNs in l-Ala-PBS or d-Ala-PBS. Credit: republished with permission of Elsevier, from Reference [52], Copyright (2020), permission conveyed through Copyright Clearance Center, Inc.

In another work, Guo et al. prepared two CMS at the molecular level by grafting l-/d-tartaric acid, namely FL MSNs and FD MSNs [52]. Apparently, nimesulide (NMS) loaded FD-MSNs and NMS loaded FL-MSNs displayed almost the same dissolution behaviour, but the cumulative release amounts were both much higher than that of pure NMS in PBS. However, more NMS released from NMS loaded FL-MSNs in l-Ala-PBS and NMS loaded FD-MSNs in d-Ala-PBS, respectively (Figure 10), which was consistent with the results of in vivo pharmacokinetics and anti-inflammatory pharmacodynamics studies. These results suggested that FL-MSNs and FD-MSNs can respond to the prepared chiral medium, and FD-MSNs performed better than FL-MSNs for the release of NMS on account of less steric hindrances. Furthermore, mesoporous silica nanoparticles with enlarged chiral pore size were synthesized aiming at the decreased drug load capacity and the increased steric hindrance of the drug release resulted from the grafting group [53]. Moreover, the properties of the modified CMS nanoparticle as drug carriers were basically investigated, demonstrating good wettability, fast degradation rate, high mucosa-adhesion ability, and long retention time [109].

### 3.5. Asymmetric Catalysis

Besides the applications mentioned above, it is also found that CMS is useful for understanding asymmetric catalysis. In the past decades, there has been tremendous progress in catalytic enantioselective synthesis, which is an effective method for synthesizing copious chiral products with only a small amount of chiral compounds [110]. CMS acted as a heterogeneous chiral trigger and proved to be effective for the enantioselective addition of diisopropylzinc to pyrimidine-5-carbaldehyde 1 due to its large reactive surface [111]. Admittedly, the application of CMS for asymmetric synthesis is still at its infancy. The outstanding features such as large surface area and thermal and mechanical stability have made them ideal candidates for asymmetric synthesis.

## 4. Concluding Remarks and Perspectives

In conclusion, this review highlighted much of the exciting progress of CMS in the past decades, including various strategies for synthesizing CMS materials, proposed mechanisms of CMS formation, and applications in different fields. The attempts on the control of morphology, pore size, and enantiomer excess have been summarized, suggesting that the reaction temperature, pH, stirring speed, and molar ratio are the common factors. Due to its unique chiral features, CMS has broadened its application in the fields of chiral separation, chiral adsorption, and drug delivery, asymmetric catalysis, etc. It can not only be used for stereoselective resolution and delivery of chiral compounds but works in the delivery of achiral drugs in chiral environments. However, in vivo biosafety and biocompatibility have hardly been investigated and involved. By and large, practical applications of chiral NPs and their assemblies are still in the initial stages. Further studies on CMS nanoparticles are needed to investigate toxicity, distribution, degradation, and accumulation in vivo, especially for those that are designed for drug delivery systems. Current studies are mainly focused on anti-inflammatory drug delivery; however, more potential applications need to be explored, including l-dopamine for Alzheimer‘s disease, cyclophosphamide for anti-tumor applications, ketamine used for anesthesia, etc. Furthermore, CMS modified with targeting agents is another promising direction for further improving therapeutical efficacy. It is believed that CMS materials could provide an important opportunity to diagnosis and therapy of various diseases as well as chiral separation and other fields.

## Figures and Tables

**Table 1:** The synthesis method, morphology, and textural properties of chiral mesoporous silica.

Synthesis Method	Morphology	Textural Properties			References
		Specific Surface Area (m^2^/g)	Pore Diameter (nm)	Pore Volume (cm^3^/g)	
Chiral anionic surfactant C_14_-l-AlaS as a template, TMAPS, or APS as CSDA.	Twisted hexagonal rod-like	600	2.20	0.37	[30]
Chiral anionic amphiphilic molecules (*N*-acyl-l-alanine) as template, TMAPS as CSDA, and various acids including HCl, HBr, HNO_3_, or H_2_SO_4_.	Twisted hexagonal rod-like	577–974	2.40–2.96	0.43–0.90	[31]
Chiral anionic surfactant C_14_-l-AlaS as a template, APTES as CSDA	Twisted-ribbon-like and various twisted-rod-like	400	3.60	0.50	[32]
Achiral CTAB as template and a chiral metal complex Λ-[Co(+)(chxn)_3_]I_3_ as contemplate.	Twisted hexagonal rod-like	1047	2.60	1.90	[33]
Chiral anionic surfactants C_14_-l/d-AlaA as template.	Twisted hexagonal rod-like	603; 735	2.20	N/A	[34]
Chiral anionic surfactant, C_14_-l/d-AlaA, and an organosilane compound, BTEE, in the presence of l/d-arginine.	Twisted hexagonal rod-like	853	2.10	0.66	[35]
Chiral anionic surfactant C_16_-l-Ala, C_16_-l-Val C16-l-Ile, and C_16_-l-Phe as templates and TMAPS as CSDA.	Twisted hexagonal rod-like	N/A	N/A	N/A	[36]
Chiral anionic surfactant *N*-acylamino acid with a bulky substituent as template and TMAPS as CSDA.	Twisted hexagonal rod-like	496–884	2.10–3.20	0.23–0.99	[37]
Chiral cationic surfactant l-4PyCl as template by sol-gel transcription in different solvents.	Helical	730	3.80	1.56	[38,39]
Chiral cationic gelators l-PhePyBr, l-ValPyBr, and l-IlePyBr as template by sol-gel transcription.	Helical, balls	354; 628	5.20; 5.00	N/A	[40,41]
Room-temperature ionic liquids 1-octadecyl-3-methylimidazolium bromide as template	Helical	893	3.270	0.99	[42]
Achiral cationic surfactant CTAB as template.	Helical	998	2.80	N/A	[43]
Achiral cationic surfactant CTAB and perfluorooctanoic acid as co-templates.	Helical rod-like	462–635	2.60	0.59–0.73	[44]
Achiral cationic surfactant CTAB as template and achiral alcohols as CSDA.	Helical	459–842	2.00–4.20	N/A	[45]
CTAB as a template and either *n*-heptanol or *n*-nonanol as CSDA.	Twisted hexagonal rod-like	930; 1329	6.83; 6.97	1.40; 2.30	[46]
Achiral cationic surfactant CTAB as template.	Helical rod-like	1027	3.40	1.02	[47]
Achiral cationic surfactant C_18_-TMS as a template in the presence of arginine, histidine, isoleucine, and proline in basic media.	Pores with an “eight-like” morphology	840–1130	2.80–3.30	0.80–1.13	[48]
Tetraethyl orthosilicate and quaternized aminosilane as silica sources together with l-phenylalanine as a chiral imprinted reagent	Irregular	730	2.30–2.50	0.47	[49]
Achiral cationic surfactant CTAB as template together with APTTES-l or APTTES-d (synthesized with APTES and l/d-tartaric acid) as chiral silica coupling agent.	Spherical	565; 387	3.30; 3.20	0.40; 0.30	[50]
Achiral cationic surfactant CTAB as template together with APTTES-l or APTTES-d (synthesized with APTES and l/d-tartaric acid) as chiral silica coupling agent.	Spherical	578; 441	3.47; 2.98	0.50; 0.33	[51]
Achiral cationic surfactant STAB as template together with APTTES-l or APTTES-d (synthesized with APTES and l/d-tartaric acid) as chiral silica coupling agent.	Spherical	571; 585	2.71; 2.74	0.69; 0.75	[52]
Achiral cationic surfactant STAB as template together with APTTES-d (synthesized with APTES and d-tartaric acid) as chiral silica coupling agent, and TMB as pore-enlarging agent.	Spherical	441; 474	2.20; 4.30	0.36; 1.10	[53]

*N*-myristoyl-l-alanine sodium salt (C_14_-l-AlaS), *N*-trimethoxysilylpropyl-*N*,*N*,*N*-trimethylammonium chloride (TMAPS), 3-aminopropyltrimethoxysilane (APS), co-structure-directing agent (CSDA), 3-aminopropyltriethoxysilane (APTES), *N*-myristoyl-l/d-alanine (C_14_-l/d-AlaA), *N*-Palmitoyl-l-Ala (C_16_-l-Ala), *N*-palmitoyl-l-Val (C_16_-l-Val), *N*-palmitoyl-l-Ile (C_16_-l-Ile), and *N*-palmitoyl-l-Phe (C_16_-l-Phe), cetyltrimethylammonium bromide (CTAB), *N*-3 [3-(trimethoxysilyl)propyl]-*N*-octadecyl-*N*,*N*-dimethylammonium chloride (C_18_-TMS), Stearyltrimethylammonium Bromide (STAB).

**Table 2:** Applications of CMS in various fields.

Applications	Compound or Drug	Key Points	Reference
Enantioselective adsorption	l- and d-alanine	With a chiral selectivity factor of 3.15	[74]
Enantioselective adsorption	l- and d-proline	l/d-proline shows different adsorption preference on CMS prepared with l-proline or d-proline	[75]
Enantioselective adsorption	Racemic valine	With a chiral selectivity factor of 5.22	[76]
Enantioselective resolution	Racemic valine and alanine	With a chiral selectivity factor of 7.52 for alanine and high enantiomeric excess of ca. 45% for valine	[77]
Enantioselective resolution	Racemic proline, isoleucine, trans-4-hydroxyproline, pipecolic acid, valine, leucine, and phenylglycine	The CMS prepared via chiral imprinting still exhibits enantioselectivity for racemic mixtures after calcination.	[48]
Stereoselective adsorption	d,l phenylalanine, d,l alanine, d,l tryptophan, d,l lysine, racemic naproxen, and racemic chlorpheniramine maleate	l-Phenylalanine imprinting exhibits enantioselective adsorption for l- and d-phenylalanine with a chiral selectivity factor of 3.24, and desirable stereoselective adsorption capacity for other racemic mixtures	[49]
Enantiomeric adsorption	*d*-(−)-Tartaric acid and *l*-(+)-Tartaric acid, *l*-valine, and *d*-Valine, as well as (+)-a-Pinene and (−)-a-Pinene	Supramolecular templated materials prepared with guanosine monophosphate (NGM-1) and folic acid have opposite enantiomeric selectivity for enantiomeric pairs	[80]
Chromatographic separations	Isomers, PAHs, linear alkanes, long-chain alkanes, Grob’stest mixture, aromatic hydrocarbons, and chiral compounds	As stationary phase for high-resolution gas chromatography separations	[89]
Chromatographic separations	Phenylalanine, tryptophan, glutamic, alanine, serine, aspartic acid, cysteine, methionine, tyrosine, and histidine	As chiral stationary phase, with hydroxypropyl-β-cyclodextrin (HP-β-CD) as the chiral selector for enantioseparation using MCE	[86]
Chiral drug delivery	Levofloxacin	In vitro sustained drug release and antibacterial activity of levofloxacin	[46]
Chiral drug delivery	Metoprolol	In vitro enantioselective controlled release	[103]
Chiral drug delivery	Ibuprofen	Release differentiation, controlled release in vitro, favorable oral bioavailability, elimination half-life, and anti-inflammatory effect in vivo.	[104,105]
Achiral drug delivery	Aspirin	In vitro controlled release	[106]
Achiral drug delivery	Indomethacin	Improved in vitro dissolution of poorly water-soluble drug	[107]
Achiral drug delivery	Curcumin	Improved in vitro dissolution of poorly water-soluble drug	[47]
Achiral drug delivery	Nimodipine	Improved in vitro dissolution; enhanced bioavailability, therapeutic effect, and brain distribution in vivo	[108]
Achiral drug delivery	Indometacin	Improved in vitro dissolution; enhanced in vivo oral bioavailability and anti-inflammatory effect	[50]
Achiral drug delivery	Indometacin	Different chiral recognition functions in the in-vitro chiral dissolution medium	[51]
Achiral drug delivery	Nimesulide	Different chiral recognition functions in the in-vitro chiral dissolution medium; enhanced in vivo oral bioavailability and anti-inflammatory effect	[52]
Achiral drug delivery	Nimesulide	Improved in vitro dissolution; enhanced bioavailability and anti-inflammatory effect in vivo	[53]
Asymmetric catalysis	Diisopropylzinc, pyrimidine-5-carbaldehyde 1	A chiral inorganic trigger of asymmetric autocatalysis	[111]
